# Transcriptome and HPLC Analysis Reveal the Regulatory Mechanisms of Aurantio-Obtusin in Space Environment-Induced *Senna obtusifolia* Lines

**DOI:** 10.3390/ijerph19020898

**Published:** 2022-01-14

**Authors:** Renjun Mao, Zhenqing Bai, Jiawen Wu, Ruilian Han, Xuemin Zhang, Weiguo Chai, Zongsuo Liang

**Affiliations:** 1 College of Life Sciences, Yan’an University, Yan’an 716000, China; mrjnwsuaf@126.com (R.M.); shanxibzq@163.com (Z.B.); wujiawende@126.com (J.W.); 2 College of Civil Engineering and Architecture, Zhejiang Sci-Tech University, Hangzhou 310018, China; hanrl@nwsuaf.edu.cn; 3 Tianjin Tasly Modern TCM Resources Co., Ltd., Tianjin 300400, China; tracymtcm@163.com; 4 Institute of Biotechnology, Hangzhou Academy of Agricultural Sciences, Hangzhou 310024, China; mrjgxyyzwy@126.com; 5 College of Life Sciences and Medicine, Zhejiang Sci-Tech University, Hangzhou 310018, China

**Keywords:** *Senna obtusifolia*, space environment-induced, secondary metabolism, anthraquinone pathway, aurantio-obtusin

## Abstract

*Senna obtusifolia* is a famous medicinal plant that is widely used in Asian countries. Its seed plays an important role in the treatment of many diseases because it contains various anthraquinones and flavonoids. Our previous studies have indicated that three space environment-induced *S. obtusifolia* lines (SP-lines) i.e., QC10, QC29, and QC46, have higher seed yield and aurantio-obtusin (AO) content. However, the underlying mechanism of higher AO content in SP-lines is still unknown. Herein, transcriptome sequencing and HPLC were employed to analyze the differences between SP-lines and ground control (GC3) and elucidate the regulatory mechanisms of AO accumulation in SP-lines. The results show that 4002 differentially expressed genes (DEGs) were identified in SP-lines versus (vs.) GC3. DEGs in the QC10 vs. GC3, QC29 vs. GC3, and QC46 vs. GC3 comparisons were classified into 28, 36, and 81 GO terms and involved in 63, 74, and 107 Kyoto Encyclopedia of Genes and Genomes (KEGG) pathways. KEGG pathway and gene expression analysis revealed that DEGs involved in anthraquinone pathways were significantly elevated in QC10 and QC46. Integrating the results of GO annotation, KEGG enrichment, and gene expression analysis, we propose that the elevated genes such as *DAHPS*, *DHQS*, and *MenB* enhance the metabolic flux in the anthraquinone pathway and promote AO content in QC10 and QC46. Taken together, this study elucidated the mechanism of AO content in SP-lines and provides valuable genetic information for *S. obtusifolia*. In addition, to the best of our knowledge, this study presents the first transcriptome analysis of environment-induced medicinal plants and paves the way to select elite *S. obtusifolia* varieties in the future.

## 1. Introduction

*Senna obtusifolia* (L.) H.S. Irwin & Barneby (synonymous with *Cassia obtusifolia*) is an annual plant that belongs to the Fabaceae family. The dried ripe seeds of *S. obtusifolia* are a rich repository of anthraquinones and flavonoids. *S. obtusifolia* seeds have been widely used in traditional medicine and herbal tea in many Asian countries, especially in India and China. Anthraquinone such as aurantio-obtusin, chrysophanol, emodin, and aloe-emodin act as the main pharmacological components of *S. obtusifolia* seeds and possess multiple medicinal properties, including anti-diabetes [1], anti-hypertension [2], anti-inflammatory [3], and liver protection activities [4]. *S. obtusifolia* seeds also exhibited significant weight loss effects [5]. In addition, *S. obtusifolia* seeds have been regarded as culinary and medicinal dual-purpose material by the National Medical Products Administration (NMPA). A recent study reported that carboxymethyl Cassia galactomannan is suitable for sustained drug delivery [6]. Because of its multiple uses, the demand for *S. obtusifolia* seeds is increasing. However, the wild *S. obtusifolia* population is almost extinct due to overcollection and habitat deterioration. Human activities also exacerbate the pollution of air and soil. Moreover, the quality of *S. obtusifolia* seeds degenerated seriously due to the long-term cultivation of a single variety. Therefore, breeding a new *S. obtusifolia* variety with high medicinal value is of great significance.

Drugs play an important role in protecting human health. The effectiveness of natural products with health potential is based on the activities of their pharmaceutical components [7,8]. Due to the high mutation rate and short breeding cycle, space environment-induced mutation breeding is emerging as a new method to breed improved varieties and to generate new genetic resources. In addition, space environment-induced mutations could produce some mutants that are difficult to obtain by conventional mutagenesis methods, such as ethylmethylsulfone (EMS) and ultraviolet-induced (UV-induced) methods. Moreover, space environment-induced mutants are valuable materials not only for breeding elite varieties but also for studying physiological processes and gene functions. For example, Huang et al. demonstrated that the slow-growing cabbage mutant is associated with chlorophyll degradation and chlorophyll metabolic processes [9]. Using an irradiation-induced mutant, Chen and Li indicated that unigenes encoding MYB, bHLH, and WD40 transcription factors were differentially expressed in the mutant, which enhanced the proanthocyanidin accumulation in the rhizomes of mutant and thus induced a higher proanthocyanidin content [10].

**Figure 1:**
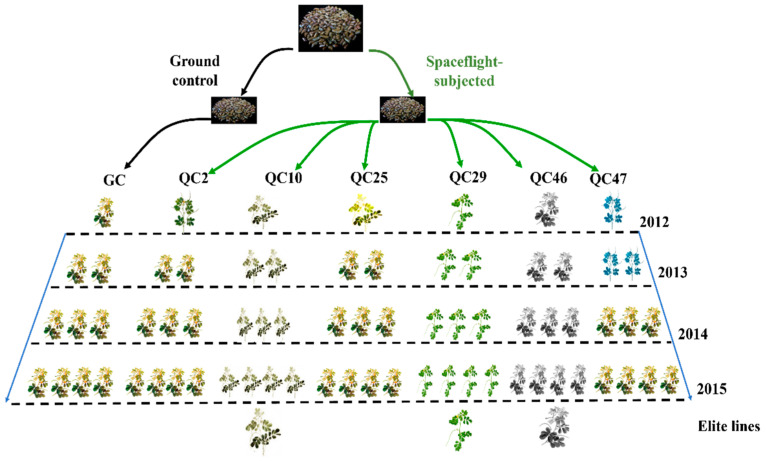
Schematic model of *S. obtusifolia* breeding program. In 2012, the first generation (SP_1_), SP-lines seeds were cultivated, and their critical biological trait was observed carefully. Several mutants were found in SP_1_ population. Seed of each line was conducted seed trait and HPLC analysis. In 2013, seeds of different lines were cultivated, and their biological trait was investigated. It was found that QC2 and QC10 cannot maintain its variation trait and exhibit the similar phenotype to GC-line. In 2014, the variation trait of QC47 was not maintained. After a three-year study, three SP-lines (QC10, QC29, and QC46) were chosen as elite lines for the further study.

In our earlier studies, to improve the germplasm resources for *S. obtusifolia* breeding applications, a space environment-induced method was employed. In total, 10 g of selected *S. obtusifolia* seeds was divided into two groups on average. One group was carried by the “ShenZhou Ⅷ” satellite into space, and the other group was stored at ground as the control (GC-line). When the satellite returned to the ground, we conducted a systematic study on the biological traits, chemical components, and antioxidant enzyme activity of space environment-induced *S. obtusifolia* lines (SP-lines). After a three-year study, we preliminarily selected three SP-lines (QC10, QC29, and QC46) with higher seed yield and aurantio-obtusin (AO) content. The breeding scheme was illustrated in Figure 1.

Transcriptome sequencing has been widely used in staple medicinal plants such as *Salvia miltiorrhiza* [11,12], *Panax ginseng* [13,14], and *Panax notoginseng* [15,16]. More than 50 transcriptome data points of *S. miltiorrhiza* (model medicinal plant) have been stored in the Sequence Read Archive (SRA, https://www.ncbi.nlm.nih.gov/sra/ (accessed on 12 May 2020)). Xu et al. offered the whole genome sequence of *S. miltiorrhiza* [17] and Ma et al. re-sequenced *S. miltiorrhiza* genome combining the latest technologies including Pacbio, Nanopore and 10X genomic to improve the assembly quality [18]. These omics data largely improved the functional gene identification and secondary metabolic pathway analysis of *S. miltiorrhiza*. Although the genome of *S. obtusifolia* has been sequenced recently [19], omics study on *S. obtusifolia*, a non-model medicinal plant, are still limited, which impedes the molecular study of *S. obtusifolia*. There is an urgent need to provide a comprehensive transcriptome analysis of *S. obtusifolia* seeds.

Although our previous studies have demonstrated that SP-lines have higher antioxidant enzyme activity and corresponding gene transcripts [20] and AO content [20,21], the underlying mechanism is still unknown. In this study, we used transcriptome sequencing combined with HPLC to (1) compare the differences between SP-lines and GC3 at the transcriptional and metabolic levels, (2) reveal the underlying mechanisms of the higher AO content in SP-lines, and (3) provide a specific transcriptome database of *S. obtusifolia* seeds. To the best of our knowledge, this study is the first attempt to evaluate the variation in space environment-induced medicinal plants by transcriptome sequencing.

## 2. Materials and Methods

### 2.1. Plant Material

Different *S. obtusifolia* seeds were cultivated in a randomized field plot at the Institute of Soil and Water Conservation, Chinese Academy of Sciences (108°07′ E, 34°29′ N, altitude 518 m). The plants received normal cultivation operations, irrigation, and weeding application. Three space environment-induced lines QC10, QC29, QC46, and the GC-line (GC1, GC2, and GC3), were cultivated in three experimental plots. On 15 July 2016, fifty seeds were collected from ten plants of each line. All samples were frozen in liquid nitrogen and stored at −80 °C until further analysis.

### 2.2. HPLC Analysis

AO content was determined via HPLC. The extraction method and processing referred to the method described by the Chinese Pharmacopoeia (2015 Edition) [22]. A Waters HPLC system equipped with a 1525 binary pump and a Waters 2487 Dual λ detector was used to analyze the extracts from *S. obtusifolia* seeds. A Waters Sunfire C_18_ column (4.6 mm × 250 mm, 5 μm) was employed for all separations. AO standard compound (111,900–201,504) was purchased from the National Institute for the Control of Pharmaceutical and Biological Products (Beijing, China).

### 2.3. RNA Extraction and Sequencing

Total RNA was extracted from six samples, i.e., QC10, QC29, QC46, GC1, GC2, and GC3, using the RNeasy Plant Mini Kit (Qiagen, Hilden, Germany) in accordance with the manufacturer’s protocol. RNA quality was verified by agarose gel electrophoresis and spectrophotometry (Thermo Scientific, NanoDrop 1000, Waltham, MA, USA). The integrity and concentration of RNA were examined via a Qubit Fluorometer (Thermo Fisher Scientific, Waltham, MA, USA) and Aligent 2100 Bioanalyzer (Aligent Technologies, Palo Alto, CA, USA). Six cDNA libraries were constructed and further sequenced by the Illumina HiSeq2000^TM^ platform.

### 2.4. Sequence Alignment and Bioinformation Analysis

The raw reads were cleaned by removing adaptor sequences, poly-N and low-quality reads. For de novo assembly, the clean reads were mapped to the contigs by Trinity [23]. Then, the contigs were assembled to construct transcripts with pair-end information and clustered to obtain unigenes. Functionally annotated with the nonredundant database (Nr, http://www.ncbi.nlm.nih.gov (accessed on 10 November 2019)), Swiss-Prot (http://www.expasy.ch/sprot (accessed on 10 November 2019)), Gene Ontology (GO, http://www.geneontology.org (accessed on 10 November 2019)), Eukaryotic Orthologous Groups (KOG, http://genome.jgi-psf.org/help/kogbrowser.jsf (accessed on 12 November 2019)), and Kyoto Encyclopedia of Genes and Genomes (KEGG, http://www.genome.jp/kegg (accessed on 15 November 2019)). KEGG pathway analysis was accomplished with the KEGG Annotation Server. Heatmap was generated by the Heml software (http://hemi.biocuckoo.org/down.php (accessed on 16 January 2020)).

### 2.5. Identification of Differentially Expressed Genes (DEGs)

DEGs were identified using DESeq software [24]. The criteria to identify DEGs were *p* < 0.05, false discovery rate (FDR) < 0.001 and log_2_Ratio > 1. We chose GC1 and GC2, which have larger raw data, to form a reference sequence. Other samples were aligned with the reference sequence. DEGs analysis was conducted between SP-lines and GC3. GO enrichment and KEGG pathway analysis of DEGs were performed using BLAST2GO software [25] and KOBAS [26].

### 2.6. Expression Analysis of Key Enzyme Genes

The expression of key enzyme genes in the anthraquinone biosynthesis pathway, including *DAHPS* (encoding 3-deoxy-7-phosphoheptulonate synthase), *DHQS* (3-dehydroquinate dehydratase), *SKM* (shikimate kinase), *ICS* (isochorismate synthase), and *MenB* (naphthoate synthase), was analyzed by quantitative real-time PCR (qRT–PCR). The relative expression levels were calculated using the 2^−ΔΔCt^ method. The detailed method was described in our previous study [20]. Briefly, qRT–PCR analysis was performed on a CFX96 Real-Time System (BioRad, USA) using SYBR Premix Ex TaqII (TaKaRa, Dalian, China) according to the manufacturer’s instructions. The PCR condition was 95 °C for 5 min, followed by 45 cycles of 95 °C for 10 s, 60 °C for 30 s, and 72 °C for 30 s. The results are shown as the mean ± standard error (SE) with six replications. Significant difference analysis was performed by two-tailed Student’s t-test, *p* < 0.05 (SPSS 23.0).

### 2.7. Protein Association Network Relationship Prediction

The protein interaction network was constructed using the method described by Mao et al. with minor modifications [27]. Briefly, five putative protein sequences (*DAHPS*, *DHQS*, *SKM*, *ICS*, and *MenB*) were submitted to the online server STRING (Version 11.5, http://string-db.org (accessed on 5 December 2020)), with the organism specified as *Arabidopsis thaliana*. The top 10 scores protein were used to construct the network.

### 2.8. Validation of DEGs by qRT–PCR

Twelve DEGs were selected for qRT–PCR analysis to verify the accuracy and reliability of transcriptome sequencing. All primers used in this study are provided in Supplementary Table S1.

## 3. Results

### 3.1. AO Content in SP-Lines and GC3

**Figure 2:**
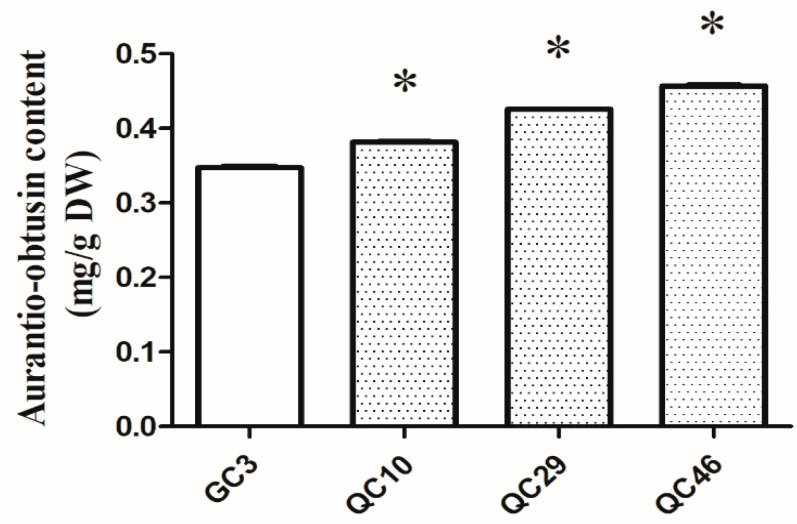
The aurantio-obtusin content of SP-lines and GC3. Note: Asterisks indicates significant differences between SP-lines and GC. (* *p* < 0.05, Student’s *t*-test). The values are representative of three biological replicates.

HPLC analysis was used to evaluate the variation in AO content in *S. obtusifolia* seeds of SP-lines and GC3. The results demonstrated that the AO content in SP-lines was significantly higher (*p* < 0.05) than that of GC3 (0.349 mg/g). Among SP-lines, QC46 had the highest AO content, reaching 0.457 mg/g, followed by QC29 (0.426 mg/g). The lowest AO content was detected in QC10 (0.382 mg/g), which was also significantly higher (*p* < 0.05) than that of GC3 (Figure 2).

### 3.2. Transcriptome Features

After removing adapters and low-quality sequences, a total of 1,011,598,554 clean reads were obtained from six libraries, containing 54.53 GB data (Table 1). The N_50_ and N_90_ for unigenes were 1905 bp and 498 bp, respectively. The average GC content was 43.87%. The error rate of sequencing was 0.02%. The counts of the unigenes in the range of 0.2–0.5 kb, 0.5–1 kb, 1–2 kb, and >2 kb were 38,035, 32,395, 22,395, and 19,412, respectively. The shortest, longest, and average lengths of the unigenes were 201 bp, 15,949 bp, and 893 bp, respectively. Transcriptome data have been deposited in the SRA database under the accession number PRJNA592774.

### 3.3. Functional Annotation and Classification

To analyze the potential function, unigenes were blasted against Nr, Nt, KO, Swiss-Prot, Pham, GO, and KOG database with the cutoff *E*-value ≤ 10^−5^. The results showed that 55,932 (49.82% of the total), 45,360 (40.40%), 21,164 (18.85%), 43,349 (38.61%), 42,845 (38.16%), unigenes hit to terms in the Nr, Nt, KO, Swiss-Prot, and Pham database, respectively. A total of 43,241 unigenes were annotated in GO database. Biological process (BP), cellular component (CC), and molecular function (MF) contained 25, 21, and 10 terms, respectively (Figure S1). The most similarity species were *Glycine max* (10,832 identified unigenes, 19.4%), followed by *Glycine soja* (8800, 15.8%), *Cicer arietinum* (6234, 11.2%), *Phaseolus vulgaris* (5477, 9.8%), and *Medicago truncatula* (4254, 7.6%). In total, 16,149 unigenes were grouped into five KEGG categories. The top three enriched pathways were ‘Carbohydrate metabolism’ (2005 unigenes), followed by ‘Translation’ (1614), and ‘Folding, sorting and degradation’ (1349). In total, 14,224 unigenes were annotated into 25 KOG categories to identify the possible functions. Detailed information of KOG and KEGG classification analysis is exhibited in Figures S2 and S3.

### 3.4. DEGs Identification between SP-Lines and GC3

**Figure 3:**
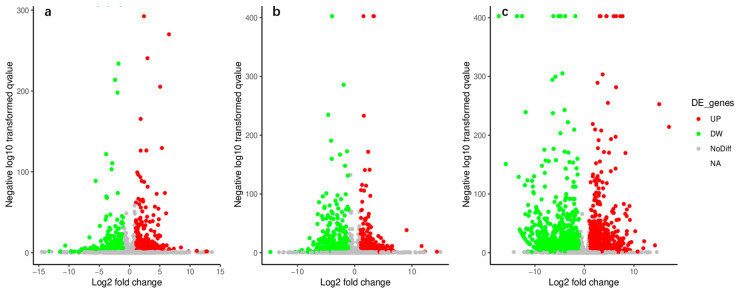
Identification of differentially expressed genes (DEGs) between QC10 vs. GC3 (**a**), QC29 vs. GC3 (**b**), and QC46 vs. GC3 (**c**). Up−, down−regulated genes were represented by red dots and green dots, respectively. The gray dots indicated there was no significant difference of genes.

Unigenes in each library were subjected to pairwise comparison analysis to identify the DEGs. The results indicated that a total of 4002 DEGs were identified in three comparisons (Figure 3). There were 487 DEGs identified in the QC10 vs. GC3 comparison, of which 297 were up-regulated and 190 were down-regulated (Figure 3a, Excel S1). QC29 vs. GC3 comparison identified 745 DEGs, including 382 up-regulated and 363 down-regulated DEGs (Figure 3b, Excel S2). A significantly larger number of DEGs (2770) were identified in the QC46 vs. GC3 comparison. Of these, 1447 and 1323 DEGs were up- and down-regulated, respectively (Figure 3c, Excel S3).

### 3.5. GO Enrichment of DEGs

**Figure 4:**
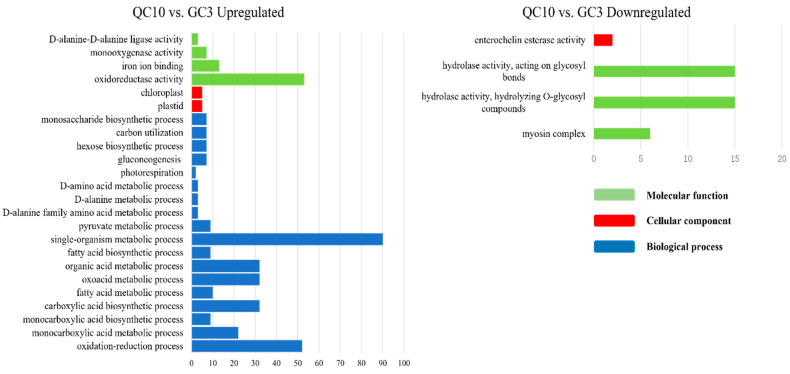
GO annotation of differentially expressed genes (DEGs) in the QC10 vs. GC3 comparison. The blue, red, and green columns represent the biological process (BP), cellular component (CC), and molecular function (MF), respectively.

**Figure 5:**
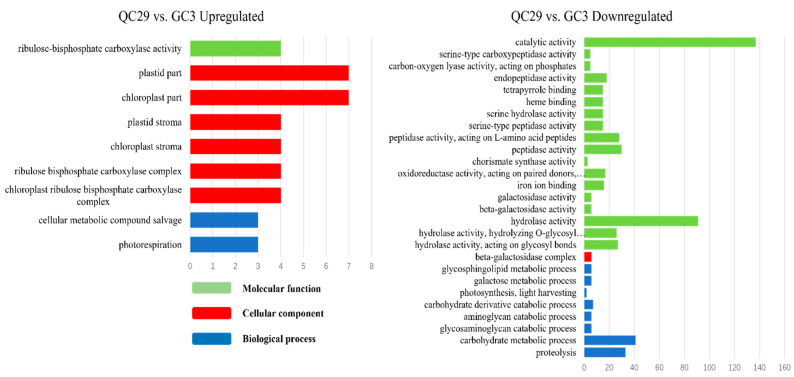
GO annotation of differentially expressed genes (DEGs) in the QC29 vs. GC3 comparison. The blue, red, and green columns represent the biological process (BP), cellular component (CC), and molecular function (MF), respectively.

**Figure 6:**
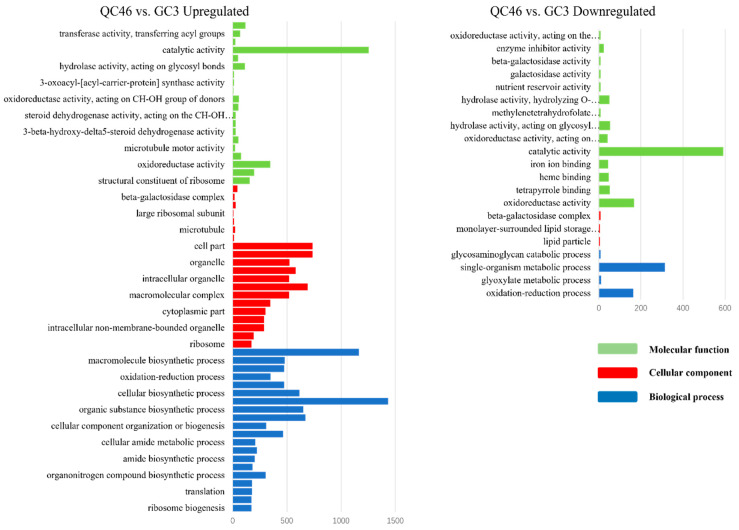
GO annotation of differentially expressed genes (DEGs) in the QC46 vs. GC3 comparison. The blue, red, and green columns represent the biological process (BP), cellular component (CC), and molecular function (MF), respectively.

To better understand the biological function of DEGs, a detailed GO enrichment analysis was performed. Large numbers of DEGs between the SP-line and GC3 were identified and functionally enriched. DEGs in the QC10 vs. GC3, QC29 vs. GC3, and QC46 vs. GC3 comparisons were classified into 28, 36, and 81 terms, respectively. In the QC10 vs. GC3 comparison, ‘Single-organism metabolic process’, ‘Oxidation-reduction process’, and ‘Oxidoreductase activity’ were dominant terms of up-regulated DEGs (Figure 4). Down-regulated DEGs were mainly enriched in ‘Hydrolase activity, acting on glycosyl bonds’ and ‘Hydrolase activity hydrolyzing O-glycosyl compound’ (Figure 4). In the QC29 vs. GC3 comparison, up-regulated DEGs were mainly involved in ‘Chloroplast part’ and ‘Plastid part’ (Figure 5). Down-regulated DEGs mainly contained ‘Catalytic activity’, ‘Hydrolase activity’, and ‘Carbohydrate metabolic process’ (Figure 5). In the QC46 vs. GC3 comparison, up-regulated DEGs were enriched in GO terms related to ‘Catalytic activity’, ‘Metabolic process’, and ‘Organic substance metabolic process’ (Figure 6), whereas the down-regulated DEGs were mainly enriched in ‘Catalytic activity’, ‘Oxidation-reduction process’, and ‘Single-organism metabolic process’ (Figure 6). These results revealed striking differences among the three comparisons in terms of GO functional terms.

### 3.6. KEGG Pathway Analysis of DEGs

**Figure 7:**
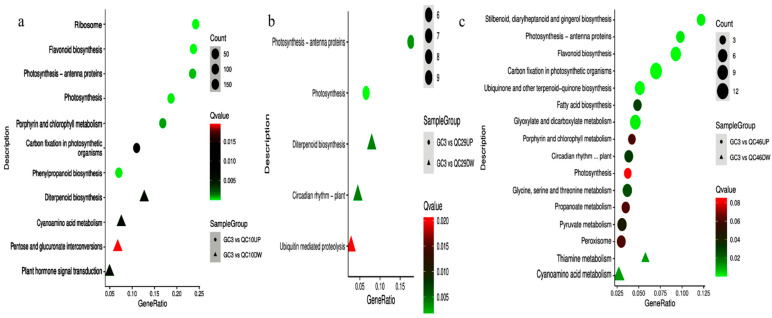
KEGG enrichment of DEGs in the comparisons of QC10 vs. GC3 (**a**), QC29 vs. GC3 (**b**), and QC46 vs. GC3 (**c**), respectively. The areas of bubbles and triangles indicate the number of enriched DEGs, while the color of bubbles indicated Q value.

To further analyze the functions of DEGs, we summarized the DEGs involved in KEGG pathways. We found that KEGG pathway enrichment was different between the SP-line and GC3. In total, 487 DEGs in the QC10 vs. GC3 comparison were annotated in 63 pathways, among which 10 pathways were significantly enriched (Figure 7a). Up-regulated pathways included flavonoid biosynthesis (ko00941), photosynthesis (ko00195), and photosynthesis-antenna proteins (ko00196). Down-regulated DEGs were enriched in cyanoamino acid metabolism (ko00460) and diterpenoid biosynthesis pathways (ko00196). A total of 745 DEGs were identified in the QC29 vs. GC3 comparison and annotated in 74 pathways, among which five pathways were significantly enriched (Figure 7b). Two pathways related to photosynthesis were enriched with up-regulated DEGs, while the other three pathways were enriched with down-regulated DEGs. Notably, 2770 DEGs in the QC46 vs. GC3 comparison were assigned to 107 pathways. Sixteen significantly enriched pathways mainly included metabolic process and synthetic process. Metabolic processes included thiamine metabolism (ko00730), propanoate metabolism (ko00640), and cyanoamino acid metabolism. Synthesis processes included flavonoid biosynthesis, fatty acid biosynthesis (ko00196), and ubiquinone and other terpenoid-quinone biosynthesis (ko04712) (Figure 7c). Cyanoamino acid metabolism and thiamine metabolism were enriched with down-regulated DEGs, while other pathways were enriched with up-regulated DEGs.

### 3.7. DEGs Involved in Anthraquinone Biosynthesis Pathway

**Figure 8:**
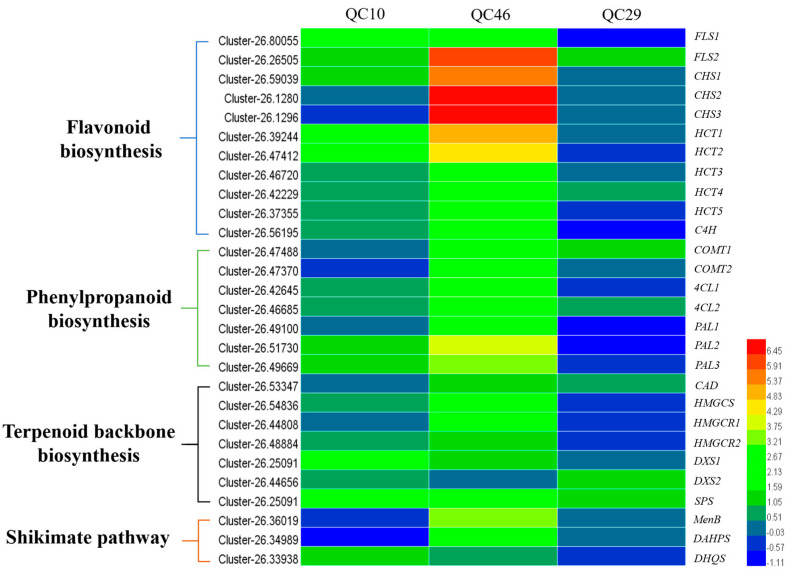
Heatmap illustration of the differentially expressed genes (DEGs) involved in anthraquinone-related pathways between SP−lines and GC3.

KEGG pathway analysis indicated that some DEGs related to secondary metabolism were significantly enriched, especially for the DEGs in the QC10 vs. GC3, and QC46 vs. GC3 comparisons. Since anthraquinone and flavonoids were the main active components in *S*. *obtusifolia* seeds, we thus focused on DEGs involved in these pathways. Anthraquinone biosynthesis shares the isochorismate pathway with phenylpropanoids and shares terpenoid backbone biosynthesis with terpenoids. In the QC10 vs. GC3 comparison, unigenes encoding *DHQS* and all-trans-nonaprenyl-diphosphate synthase (*SPS*) showed 1.52- and 2.08-fold increases (Figure 8). In the QC46 vs. GC3 comparison, unigenes encoding hydroxy-methylglutaryl-CoA synthase (*HMGCS*, Cluster-26.54836) and hydroxy-methylglutaryl-CoA reductase (*HMGCR*, Cluster-26.44808, Cluster-26.48884) showed 2.27-, 2.03- and 1.57-fold increases, respectively (Figure 8). Notably, the expression of *DAHPS* and *MenB*, two key enzyme genes in the shikimate acid pathway, increased 3.23- and 3.34-fold in QC46, respectively.

Flavonoids are another important secondary metabolite in *S. obtusifolia* and are tightly associated with anthraquinone metabolism. Several genes encoding the enzymes involved in flavonoid biosynthesis were found to be highly expressed in QC10 and QC46, including 4-coumarate-CoA ligase (*4CL*), cinnamyl-alcohol dehydrogenase (*CAD*), *C4H*, and caffeic acid 3-O-methyltransferase (*COMT*). Five shikimate O-hydroxycinnamoyl transferase genes (*HCTs*, Cluster-26.39244, Cluster-26.47412, Cluster-26.46720, Cluster-26.42229 and Cluster-26.37355) were up-regulated, among which Cluster-26.39244 was the most differentially expressed (4.85-fold higher) (Figure 8). Noticeably, in the terpenoid backbone biosynthesis pathway (ko00900), no unigenes were identified as DEGs except Cluster-26.44656 in the QC29 vs. GC3 comparison. These results indicated that the transcription levels of enzyme genes in anthraquinone biosynthesis pathways were enhanced in QC10 and QC46.

### 3.8. Expression Analysis of Key Enzyme Genes in the Shikimate Acid Pathway

**Figure 9:**
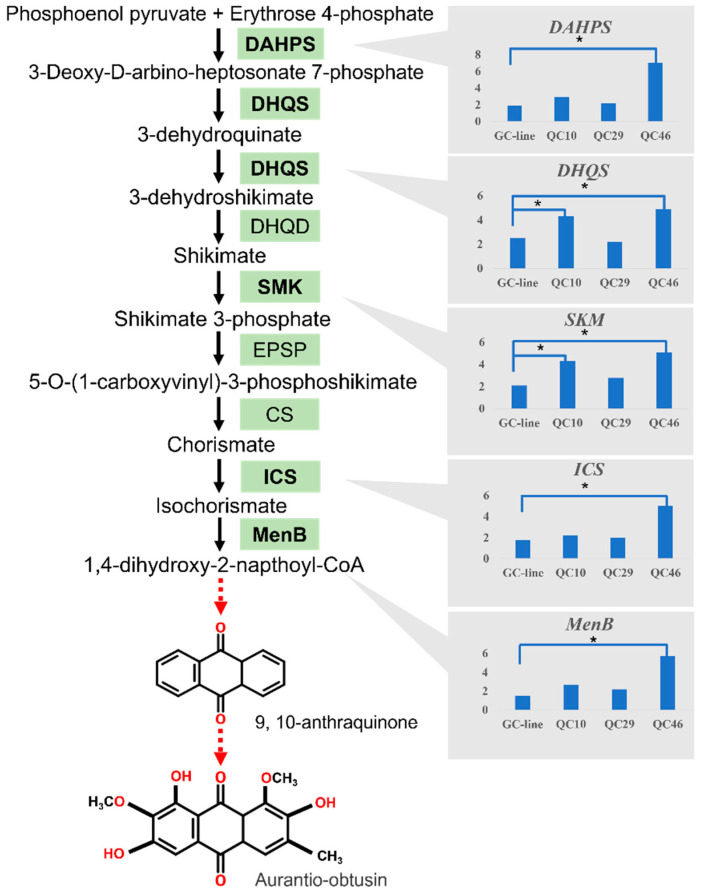
Expression profiles of transcripts encoding enzymes involved in the shikimate pathway. The genes measured by qRT–PCR are marked in bold. Solid arrows represent one-step reaction, and red arrows represent multiple-step reactions. The results were shown as the mean ± standard error (SE) with six replications. Asterisks indicate statistically significant differences (*p* < 0.05, Student’s *t*-test). *DAHPS*, 3-Deoxy-7-phosphoheptulonate synthase; *DHQS*, 3-Dehydroquinate synthase; DHQD, 3-Dehydroquinate; *SMK*, shikimate synthase; EPSP, EPSP synthase; CS, Chorismate synthase; *ICS*, isochorismate; *MenB*, naphthoate synthase.

The shikimate acid pathway is one of the upstream pathways of anthraquinones biosynthesis. To further study the variations of anthraquinones pathway genes of SP-lines, we selected five identified genes in the shikimate acid pathway to perform the expression analysis to clarify the direct reason for the increase of AO content in SP-lines (Figure 9). For QC10 and QC46, the expression of *DAHPS* showed 1.53- and 3.84-fold up-regulation which were significantly higher (*p* < 0.05) than that of GC3. Similarly, *DHQS* showed 1.72- and 1.96-fold up-regulation. In addition, the expression of *SKM* was also remarkably up-regulated which was 2.05- and 2.43-fold to that of GC3. Notably, significantly higher (*p* < 0.05) expression of *ICS* and *MenB* was only detected in QC46, which was 2.80- and 3.80-fold higher than that of GC3 (Figure 9). These results demonstrated that the key enzyme genes involved in anthraquinone pathways were more highly expressed in QC10 and QC46. No remarkable variation of those genes was detected in QC29. The gene expression patterns of QC10 and QC46 were consistent with their higher AO contents. To further analysis the relationship between gene expression level and AO content, Pearson correlation was employed to conduct correlation analysis. The results indicated that the expression level of these five genes have a positive correlation with AO content, among which *DAHPS* and *SKM* have higher correlation value (Table S2).

### 3.9. Protein Interaction Network Analysis

**Figure 10:**
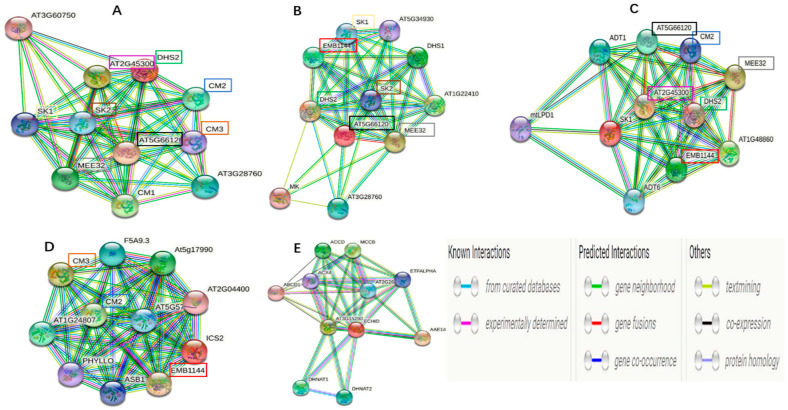
Protein interaction network predictions of *DAHPS* (**A**), *DHQS* (**B**), *SKM* (**C**), *ICS* (**D**), and *MenB* (**E**) based on orthologs in *Arabidopsis thaliana*. The network was predicted by STRING (Version 11.5, http://string-db.org (accessed on 5 December 2020)).

Five key enzymes involved in anthraquinones biosynthesis were subjected to predicted interaction partner analysis (Figure 10). The best three closely linked interaction partners to DAHPS included AT5G66120 (catalyzes the second step in the shikimate pathway), CM1 (encodes chorismate mutase), and AT2G45300 (catalyzes the transfer of the enolpyruvyl moiety of phosphoenolpyruvate) was the most relevant (0.997). *DHQS* exhibited a close functional partnership with DHS2 (phospho-2-dehydro-3-deoxyheptonate aldolase 2) and MEE32 (bifunctional 3-dehydroquinate dehydratase/shikimate dehydrogenase). Furthermore, *ICS* was associated with two close functional partners: EMB1144 (chorismate synthase) and CM3 (chorismate mutase 3). SKM was most closely linked to AT2G45300, followed by MEE32. The most useful predicted partners for *MenB* were AAE14 (2-succinylbenzoate-CoA ligase) and AT3G15290 (3-hydroxybutyryl-CoA dehydrogenase-like protein). *MenB* was also closely linked to DHNAT2 (catalyzes the hydrolysis of the thioester bond of 1,4-dihydroxy-2-naphthoyl-CoA in peroxisomes).

### 3.10. Transcriptome Validation

**Figure 11:**
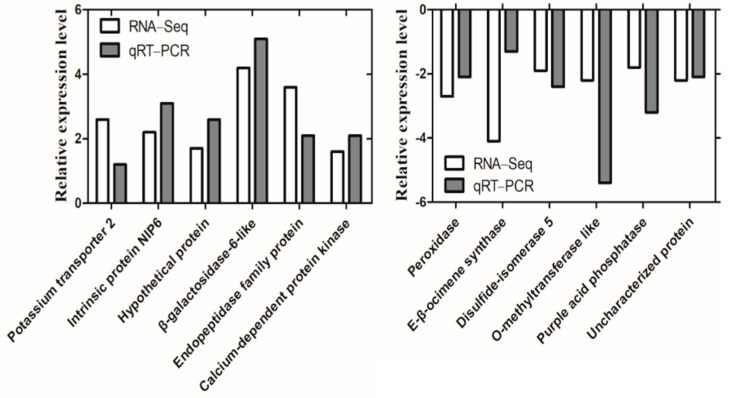
Twelve differentially expressed genes (DEGs) were validated by qRT−PCR. DEGs detected by transcriptome and qRT−PCR were present in white and gray columns, respectively.

To validate the results of transcriptome sequencing, 12 DEGs were selected to perform qRT−PCR analysis. The expression patterns of selected DEGs showed the same variation tendency in the transcriptome and qRT−PCR with different fold-changes (Figure 11). For example, potassium transporters showed a 2.6-fold increase in transcriptome analysis, while qRT−PCR exhibited a 1.2-fold increase. Similarly, transcriptome analysis showed that beta-ocimene synthase and purple acid phosphatase were 4.1- and 1.8-fold down-regulation, while qRT−PCR results were 1.3- and 3.2-fold down-regulation. Correlation analysis of transcriptome and qRT−PCR results exhibited that they were highly correlated (R^2^ = 0.7887), indicating the reliability and accuracy of transcriptome analysis.

## 4. Discussion

Owing to its low cost and high efficiency, transcriptome sequencing has been widely used to elucidate secondary metabolite pathways of medicinal plants [28,29,30]. For medicinal plants, specific secondary metabolites and related genes have attracted much attention. The combination of transcriptome and HPLC could provide a comprehensive analysis at both the transcriptional and metabolic levels and offer valuable information; thus, it has been used in *Zanthoxylum bungeanum* [31], *Stevia rebaudiana* [32] and *Sophora flavescens* [33]. In this study, we used transcriptome and HPLC to compare the differences between the SP-lines and GC3 at the transcriptional, metabolite, and omics levels, aiming to explore the underlying mechanisms of AO accumulation in SP-lines and laying a foundation for breeding new *S. obtusifolia* varieties.

### 4.1. Secondary Metabolites in Space Environment-Induced Medicinal Plants Were Altered Significantly

Space environment-induced medicinal plants showed significant variation in their secondary metabolites. A previous study on space environment-induced *Glycyrrhiza uralensis* indicated a significant increase in glycyrrhizin and glycyrrhizic acid contents, which were 1.14- and 2.42-fold to that of the control plant [34]. Likewise, a study on *S. miltiorrhiza* demonstrated a remarkably higher phenolic acid content in space environment-induced materials [35]. In this study, QC29 and QC46 showed significantly higher AO content than GC3. AO is a specific secondary metabolite of *S. obtusifolia* and mainly accumulates in its seeds [36,37]. Additionally, AO is a quality marker of *S. obtusifolia* seeds and has great pharmaceutical value. The latest studies indicated that AO has anti-inflammatory [38] and antiviral properties [3] and could ameliorate lung inflammation [39]. SP-lines have great potential to become elite varieties with high AO and deserve further study.

### 4.2. De Novo Assembly of S. obtusifolia Seeds Transcriptome

We performed a comprehensive analysis of *S. obtusifolia* seeds and constructed a high-quality transcriptome database. More than 101 million reads with 54.53 GB data were obtained. Considering that studies on the molecular aspect of *S. obtusifolia* are scarce, these extensive transcriptome data will provide abundant information for further molecular study of *S. obtusifolia*. Amino acid similarity analysis indicated that the top five similarity species were *Glycine max*, *Glycine soja*, *Cicer arietinum*, *Phaseolus vulgaris*, and *Medicago truncatula*, which all belong to the Leguminosae family. *Glycine max* and *Medicago truncatula* are model plants and have been thoroughly studied. Because of high amino acid similarity, further study on *S. obtusifolia* may refer to these two model species.

### 4.3. SP-Lines Have Different AO Accumulation Mechanisms

Secondary metabolites such as flavonoids, anthraquinones, and alkaloids from medicinal plants are receiving increasing attention due to their remarkable physiological functions and pharmaceutical value. SP-lines have a higher AO content, which is a desired trait of breeding. Nevertheless, we are also interested in elucidating the underlying mechanism of AO accumulation. GO analysis of DEGs indicated that GO terms in three comparisons were quite different. Unexpectedly, no GO term was enriched in any comparisons. Enriched GO terms differed significantly in three comparisons, suggesting the different transcriptional patterns of the three SP-lines.

In an earlier study, based on experimental results and literature reports, we speculated that peroxidase (*POD*) and ascorbate peroxidase (*APX*) are involved in regulating AO accumulation and lead to a higher AO content in QC29 [20]. Here, transcriptome results indicated that 12 unigenes were identified as antioxidant enzyme genes, among which three *POD* (Cluster-26.44407, Cluster-26.33814, Cluster-26.46919) and two *APX* (Cluster-26.50084, Cluster-26.49222) genes were identified as DEGs in QC29. However, no DEGs were identified as *APX* in QC10 and QC46. These results supported our hypothesis that higher AO content in the QC29 is associated with *POD* and *APX* regulation. On the other hand, DEGs in the QC29 vs. GC3 comparison were enriched in only five KEGG pathways (Figure 7b). However, DEGs in the QC10 vs. GC3, and QC46 vs. GC3 comparisons were enriched in 10 and 16 KEGG pathways, respectively, most of which were secondary metabolism pathways (Figure 7a,c). These findings led us to pay more attention to the DEGs enriched in the secondary metabolism pathway, especially the anthraquinone pathway.

The expression pattern of DEGs in anthraquinone pathways was also diverse among the three comparisons. Unigenes coding *DHQS*, *HCT*, and *SPS* were dramatically up-regulated in QC10, and unigenes encoding *DAHPS*, *HMGCS*, *HMGCR*, and *HCT* were up-regulated in QC46. Notably, no unigenes encoding key enzyme genes were detected as DEGs in QC29. These results indicated that up-regulated key enzyme genes increased enzyme activity and metabolic flux and consequently promoted AO content in QC10 and QC46. In general, the expression pattern of enzyme genes in metabolic pathways has significant effects on metabolite accumulation. Many studies have demonstrated that the enhanced expression of key enzyme genes in the biosynthetic pathway can improve the secondary metabolite content. For example, a study on *S. miltiorrhiza* demonstrated that overexpression of *SmANS* significantly enhanced the anthocyanin content by inhibiting phenolic acid biosynthesis [40]. Similarly, the up-regulation of *4CL3* resulted in a 4.6-fold increase in lignin content in *Isatis indigotica* hair roots [41]. These studies showed that the up-regulation of key enzyme genes leads to an increase in metabolic flux, resulting in an increase in the specific metabolite content.

In contrast to the fact that all the functional genes encoding key enzymes were identified in *S. miltiorrhiza* [42,43,44,45], no functional gene was identified in *S. obtusifolia*, and few reports about the anthraquinone pathway have been published. Anthraquinone biosynthesis takes place mainly through terpenoid backbone biosynthesis/shikimic acid pathways, but the complete biosynthetic pathway of AO remains unclear [46,47]. Therefore, we detected the expression level of five identified genes in the shikimate acid pathway to reflect the variation pattern of the anthraquinone pathway. As expected, these five genes were more highly expressed in SP-lines, especially in QC46. The expression levels of *DAHPS*, *DHQS*, and *SKM* were also significantly enhanced in QC10. No significant variation in these genes was detected in QC29. Pearson correlation analysis indicated that the expression levels of key enzyme genes were positively associated with AO content, especially *DAHPS* and *SKM*. The results indicated that the expression levels of enzyme genes in the anthraquinone pathway were significantly varied. Based on protein interaction analysis and function annotation in STRING, we proposed that CM2, MEE32, and DHS2 may play important role in AO biosynthesis and should be experimentally determined in next study. Integrating the results of GO annotation, KEGG pathway and gene expression analysis, we proposed that QC10 and QC46 accumulated higher AO content mainly related to the up-regulated gene such as *DAHPS*, *DHQS*, and *MenB* in the anthraquinone pathway, while *POD* and *APX* may function as the signal molecular to participate in AO accumulation in QC29. Further molecular studies on DEGs in the anthraquinone biosynthesis are needed to identify the functions of these candidate genes. The corresponding mechanism of the higher AO content in QC29 remains to be further studied.

## 5. Conclusions

AO is a specific secondary metabolite of *S. obtusifolia* and has great pharmacological value. Investigating the underlying mechanism of AO biosynthesis is of great significance. In this study, we performed transcriptome and HPLC analyses of *S. obtusifolia* seeds and characterized the differences in transcriptional and AO content between SP-lines and GC3. There were significant differences in the expression level of AO biosynthesis pathway genes among three SP-lines. Based upon the results of transcriptome and gene expression analysis, it can be concluded that the high expression of genes in the anthraquinone pathway resulted in a significantly higher AO content in QC10 and QC46. In addition, these transcriptome data will be useful for elucidation of the anthraquinone biosynthesis pathway and for breeding elite *S. obtusifolia* varieties in the future.

## Figures and Tables

**Table 1:** Summary of transcriptome data of *S. obtusifolia* seeds.

Sample	Raw Reads	Clean Reads	Clean Bases (GB)	Error Rate (%)	Q20 (%)	Q30 (%)	GC Content (%)
GC1	62,851,716	60,127,312	9.02	0.02	97.10	92.69	43.75
GC2	61,381,444	58,745,742	8.81	0.02	97.29	93.06	43.64
GC3	60,908,612	58,019,204	8.70	0.02	97.13	92.75	44.22
QC10	73,498,576	71,952,222	10.79	0.02	96.58	91.40	43.82
QC29	56,322,732	55,073,530	8.26	0.02	96.79	91.86	43.58
QC46	61,461,010	59,680,544	8.95	0.02	95.87	89.94	44.18

GC1: Ground control sample 1. QC10, QC29 and QC46 are three space environment-induced samples.

## Data Availability

The transcriptome data used in this study were uploaded to the National Center for Biotechnology Information (NCBI) SRA database under the accession number PRJNA592774.

## Supplementary Materials

The following supporting information can be downloaded at: https://www.mdpi.com/article/10.3390/ijerph19020898/s1, Figure S1: Gene Ontology (GO) classification of the assembled unigenes from the *S. obtusifolia* transcriptome. Unigenes were classified into molecular function (MF), biological process (BP), and cellular component (CC) category; Figure S2: Functional classification of KOG analysis; Figure S3: Functional classification of KEGG analysis. A: Cellular processes; B: Environmental information processing; C: Genetic information processing; D: Metabolism; E: Organismal systems; Table S1 Primers used in this study; Table S2 Pearson correlation coefficients between gene expression levels and aurantio-obtusin content. Excel S1: DEGs identified in the QC10 vs. GC3 comparison; Excel S2: DEGs identified in the QC29 vs. GC3 comparison; Excel S3: DEGs identified in the QC46 vs. GC3 comparison.
