# Supplementary material for: Transcriptome and HPLC Analysis Reveal the Regulatory Mechanisms of Aurantio-Obtusin in Space Environment-Induced Senna obtusifolia Lines
Source: Int J Environ Res Public Health. 2022 Jan 14;19(2):898. doi: 10.3390/ijerph19020898 (PMC8776150; doi:10.3390/ijerph19020898)
Supplement: Supplementary file 1 [file ijerph-19-00898-s001.zip › R-Supplementary Figures and Tables..pdf]

International Journal of
Environmental Research and Public Health

MDPI

Supplementary Materials: The following supporting information can be downloaded at: www.mdpi.com/xxx/s1, Figure S1: Gene Ontology (GO) classification of the assembled unigenes from the *S. obtusifolia* transcriptome. Unigenes were classified into molecular function (MF), biological process (BP), and cellular component (CC) category; Figure S2: Functional classification of KOG analysis; Figure S3: Functional classification of KEGG analysis. A: Cellular processes; B: Environmental information processing; C: Genetic information processing; D: Metabolism; E: Organismal systems; Table S1 Primers used in this study; Table S2 Pearson correlation coefficients between gene expression levels and aurantio-obtusin content. Excel S1: DEGs identified in the QC10 vs. GC3 comparison; Excel S2: DEGs identified in the QC29 vs. GC3 comparison; Excel S3: DEGs identified in the QC46 vs. GC3 comparison.

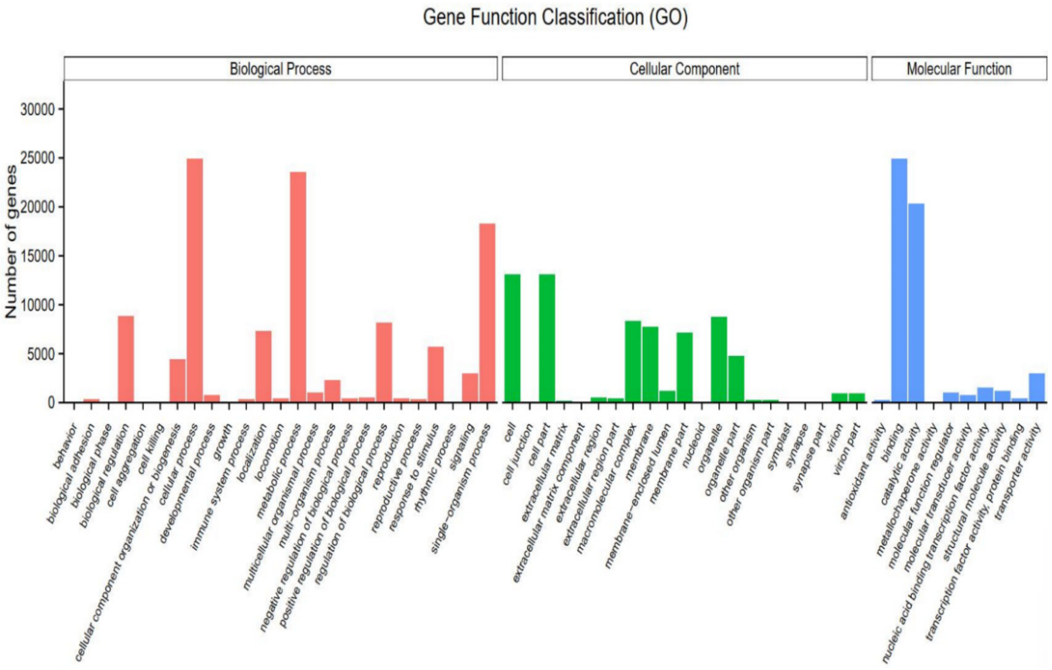

Figure S1. Gene Ontology (GO) classification of the assembled unigenes from the *S. obtusifolia* transcriptome. Unigenes were classified into molecular function (MF), biological process (BP), and cellular component (CC) category.

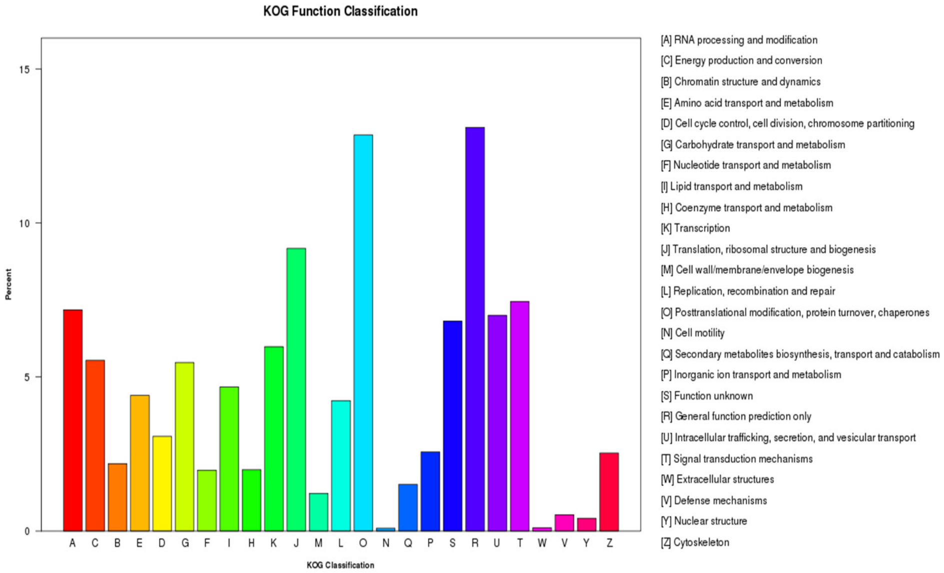

Figure S2. Functional classification of KOG analysis.

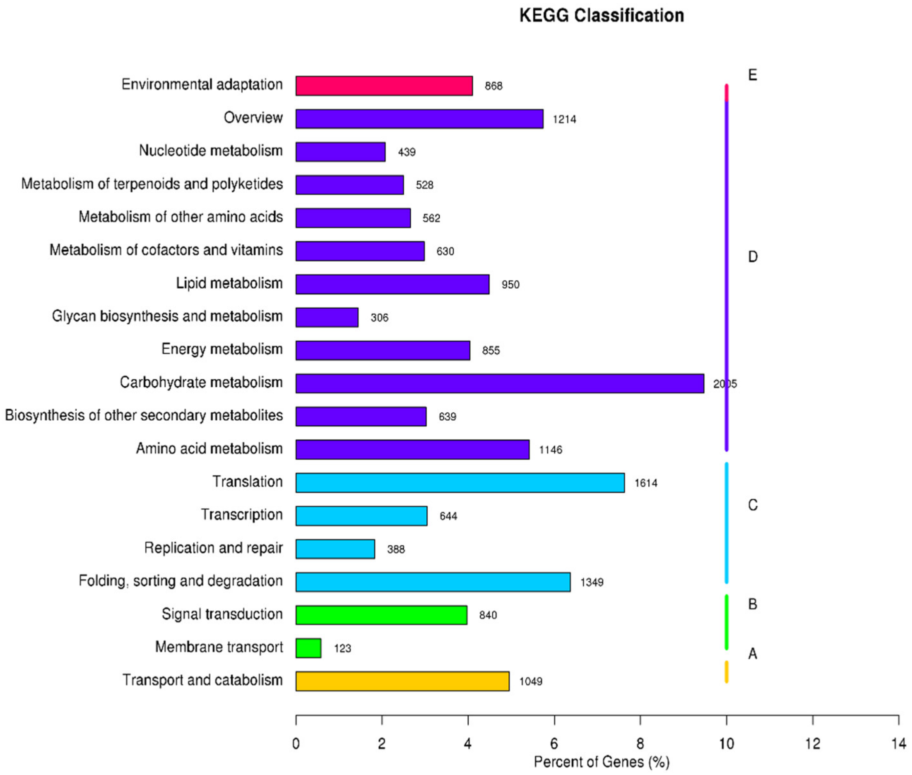

Figure S3. Functional classification of KEGG analysis. A: Cellular processes; B: Environmental information processing; C: Genetic information processing; D: Metabolism; E: Organismal systems.

Table S1. Primers used in this study.

Genes	Primer 5′-3′ Forward	Primer 5′-3′ Reverse	Usage
Potassium transporter	ATGGCAAGTGTTGGGACACATCA	ATGGCACCAATGTCAGAGTCCAG	Transcriptome validation
Intrinsic protein	ACTTGCTGCCATGTGGAAGACTG	ATGCCAAACACAGCACACTACCG	
Hypothetical protein	ATCCTCGAAGCACTCCTCAGATG	ACTGTCCAGGTTGAGGCTCATGT	
Beta-galactosidase	AGCCACCCATGAAGTCATCTGGT	TGTGGATGCGTACGAGAGATCAA	
Endopeptidase protein	TCCACGGCATCTTCTTCCAAGCT	GTCTAGGAGACATGAGGCGATCA	
Calcium-dependent	ATGTGAGCTCAAAGGCAACATCT	ATGCGTTTGTAACCACAGCCTCA	
Peroxidase	AGGTACCTGCTCCTCCTAAAGCT	TCATACAACTCGTAGTGTCACCG	
Beta-ocimene synthase	AAGGAGGCTGCTCGAACTTCAGT	ATTGATGAAGCGGGAAGCAAAGA	
Disulfide-isomerase	TTCTTCTACTTTCACTCCGGTGC	TGGAGGTGAGTCCAGGGATTCAG	
Methyltransferase-like	ATACACGCGTGCTTTGATGC	GGCCACCTTCACCATTCAGA	
Uncharacterized	ATCTGTGGCATTGCATCCATCCA	AGCTTCAATAGGAGCTGGGAACA	
Purple acid phosphatase	AGTTGCCACACATAAATGGCTGT	TGCATTTACCCCAGCAGCTATGA	
DAHPS	ACCACCACCACTCTGAAAATGGC	GGTGAAGAGGGATGGAAATGACG	Gene expression

DHQS	CCATCAGCAACCTTCTTATCCAC	TGTGAAGCGAGTTCATGCCATTC	analysis
SKM	ATACACGCGTGCTTTGATGC	GGCCACCTTCACCATTCAGA	
ICS	ATGCCAAACGCAACGAATGAA GC	TGTGAGTGGAGCCAATCCATTGC	
MenB	GCGCCTTCGCCTTCTTTATGTTA	AAGCATTTCTTCTCTCTGGCCTA	
18S RNA	TTGAAGAACGGTGATGCAGGTA	CACACTCTTGATGACTCCCACA	

Table S2. Pearson correlation coefficients between gene expression levels and aurantio-obtusin (AO) content.

Variable	***DAHPS***	***DHQS***	***SKM***	***ICS***	***MenB***	**AO**
DAHPS	1	0.750	0.819	0.996**	0.956 *	0.936*
DHQS	0.750	1	0.933*	0.784	0.766	0.640
SKM	0.819	0.933 *	1	0.819	0.977*	0.901*
ICS	0.996**	0.784	0.819	1	0.730	0.827
MenB	0.956 *	0.766	0.977*	0.730	1	0.792
AO	0.936*	0.640	0.901*	0.827	0.792	1

Significance levels at $p < 0.05$ (*) and $p < 0.01$ (**).
